# Causal relationship between asthma and ankylosing spondylitis: A bidirectional two-sample univariable and multivariable Mendelian randomization study

**DOI:** 10.1515/med-2025-1177

**Published:** 2025-07-08

**Authors:** Yiming Yao, Shiqiang Zhou, Yumin Fang, Wenxing Zeng, Shaofeng Zhan

**Affiliations:** Department of Respiratory and Critical Care Medicine, Guangzhou University of Chinese Medicine, Guangzhou, Guangdong Province, 510000, China; Department of Traumatology, Guangzhou University of Chinese Medicine, Guangzhou, Guangdong Province, 510000, China; Department of Respiratory and Critical Care Medicine, Guangzhou University of Chinese Medicine, 16 Jichang Road, Baiyun District, Guangzhou, Guangdong Province, 510000, China

**Keywords:** asthma, eosinophilic asthma, ankylosing spondylitis, Mendelian randomization, causal relationship

## Abstract

**Background:**

Previous research has indicated a possible association between asthma and ankylosing spondylitis (AS).

**Methods:**

We pinpointed single nucleotide polymorphisms linked to various forms of asthma and AS, employing them as instrumental variables for a bidirectional two-sample Mendelian randomization (TSMR) analysis. Our TSMR analysis focused on European populations to minimize racial confounding. Multivariate adjustments for body mass index (BMI), smoking, and alcohol use were performed to control for confounders. Colocalization analysis was used to validate MR findings and explore genetic links between asthma and AS.

**Results:**

Individuals with asthma and eosinophilic asthma exhibited a relatively higher risk of AS (asthma: OR 1.31, 95% CI 1.07–1.62, *P* = 0.008; eosinophilic asthma: OR 1.24, 95% CI 1.005–1.544, *P* = 0.044). Allergic asthma, childhood-onset asthma, and obesity-related asthma showed no causal relationship with AS (allergic asthma: IVW *P* = 0.27; childhood-onset asthma: IVW *P* = 0.66; obesity-related asthma: IVW *P* = 0.53). After adjusting for cigarette smoking, alcohol consumption, and BMI, the results supported a direct causal effect of asthma on the increased risk of AS onset (IVW *P* = 0.001).

**Conclusion:**

This study revealed that a positive causal connection between asthma, specifically eosinophilic asthma, and AS.

## 1 Introduction

Asthma is a diverse condition often marked by persistent inflammation of the airways [1]. Ankylosing spondylitis (AS) is a chronic inflammatory disease with a multifaceted etiology influenced by genetic factors, demographic characteristics (such as age of onset, gender, race, and family history), and environmental influences [2]. AS primarily affects the mid-axial and sacroiliac joints, but extra-articular features can also be present. AS can affect the tracheobronchial tree and lung parenchyma, leading to various pulmonary manifestations [3,4]. Zhou et al. performed a Mendelian randomization (MR) study that verified a positive causal link between asthma and rheumatoid arthritis [5]. The exact pathogenesis of AS remains unclear, with its inflammatory and immunological mechanisms still under debate [6]. A retrospective cohort study by Shen et al., conducted in a Taiwanese population, revealed that individuals with AS had a 1.74-fold higher prevalence of asthma compared to those without AS [7]. However, no relevant cohort study has confirmed the effect of asthma on AS. Given the constraints of cohort studies, additional research is required to establish this connection. Additionally, studies examining the genetic association between asthma and AS have not yet been conducted. MR is a commonly applied epidemiological approach to investigate causal links between exposures and outcomes [8–10]. By utilizing genetic variants as an instrumental variable (IV), MR studies effectively minimize the influence of reverse causation and confounding factors. In order to overcome the shortcomings of existing research, we conducted a bidirectional two-sample Mendelian randomization (TSMR) analysis, focusing on European populations, to investigate the potential causal link between asthma, including its subtypes and AS. This study aims to determine the direction of causality and offer further insights for etiological research on AS.

## 2 Materials and methods

### 2.1 Study design

**Figure 1:**
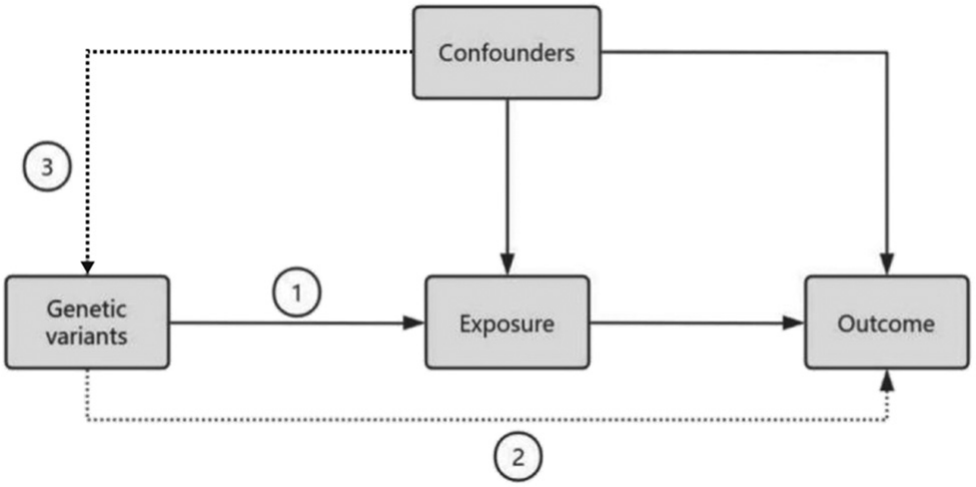
The rational of Mendelian randomization: 1 represents the IVs that are strongly associated with the exposure; 2 indicated IVs must influence the outcome only through the exposure; 3 shows that the IVs must not associated with the con-founders.

Initially, we performed a TSMR analysis in both directions to assess the causal link between asthma, its various forms, and AS. We employed genetic variations (single nucleotide polymorphisms, SNPs) identified from existing literature or genomic reference groups to assess the possible causal impact of asthma on AS. We conducted multivariate analyses to account for potential confounders like body mass index (BMI), smoking, and alcohol using frequency, ensuring that these causal effects were not influenced by these factors. For an MR analysis to be valid, three main conditions must be met: (1) genetic variants must be closely linked to the exposure variables, (2) the exposure variables and outcomes should not be affected by any known confounders, and (3) genetic variants should not impact the outcomes through routes other than the exposure being investigated. The relationship between exposure and outcome is illustrated in Figure 1.

### 2.2 Data source

The study investigated exposure factors associated with AS and asthma, including asthma, allergic asthma, childhood-onset asthma, obesity-associated asthma, and eosinophilic asthma. Genome-wide association study (GWAS) data for these exposure factors were retrieved from the FinnGen consortium (https://www.finngen.fi/fi). Additionally, data on BMI, smoking habits, and alcohol consumption frequency were obtained from the IEU OpenGWAS database (https://gwas.mrcieu.ac.uk/) to perform multivariable analyses. Comprehensive details regarding the exposure and outcome datasets are summarized in Table 1 for reference.

**Table 1:** Information of the exposure and outcome datasets

GWAS ID	Exposure or outcome	Resource	Participants included in analysis
finngen_R10_J10_ASTHMA_EXMORE	Asthma	FinnGen	219734 European-descent individuals
finngen_R10_M13_ANKYLOSPON	Ankylosing spondylitis	FinnGen	294770 European-descent individuals
finngen_R10_ALLERG_ASTHMA	Allergic asthma	FinnGen	228085 European-descent individuals
finngen_R10_ASTHMA_CHILD_EXMORE	Childhood asthma (age < 16)	FinnGen	219734 European-descent individuals
finngen_R10_ASTHMA_EOSINOPHIL_SUGG	Suggestive for eosinophilic asthma	FinnGen	365497 European-descent individuals
inngen_R10_ASTHMA_OBESITY	Obesity-related asthma	FinnGen	365497 European-descent individuals
ukb-a-248	BMI	IEU GWAS	336107 European-descent individuals
ukb-a-25	Alcohol intake frequency	IEU GWAS	336965 European-descent individuals
ukb-a-236	Ever smoked	IEU GWAS	336067 European-descent individuals

### 2.3 Selection of IVs

MR studies employ IVs to investigate the causal links between exposure factors and outcomes [11]. IVs are typically genetic variants, with SNPs being the most common. To guarantee the strength and dependability of our MR analysis, we adopted a stringent method for choosing IVs. Initially, we pinpointed SNPs that were significantly linked to the exposure (*P* < 5 × 10^−6^). Second, we removed linkage disequilibrium (LD) among SNPs to avoid bias, ensuring *r*² < 0.001 and a clumping distance of 10,000 kb. *F*-statistics were used to confirm a robust link between IVs and exposure, with values above 10 typically indicating a strong association. According to these standards, 143 SNPs linked to asthma, 34 to AS, 55 to childhood-onset asthma, 48 to obesity-related asthma, 67 to allergy-related asthma, and 54 to eosinophilic asthma were employed as IVs for further examination. Each of these IVs had *F* values exceeding 10, suggesting that any bias in these IVs did not directly influence the evaluation of causal relationships.

### 2.4 Statistical analysis

Every analysis was conducted with the TwoSampleMR library (v0.5.11) in R software (v4.3.3). Once suitable exposure SNPs were identified, we primarily employed inverse variance weighting (IVW) regression to evaluate causality, as the IVW method is widely regarded as the most effective for uncovering causal relationships in TSMR studies [12]. Subsequently, we performed further MR analyses utilizing methods such as MR-Egger, weighted median, weighted mode, and simple mode. A *P*-value of less than 0.05 was considered suggestive evidence of a potential association. Odds ratios (ORs) and standard errors (SEs) were calculated to indicate effect sizes. Furthermore, IVW regression and MR-Egger regression were used to assess heterogeneity, with Cochran’s *Q*-test quantifying the heterogeneity [13]. MR-Egger regression was also applied to determine the possibility of pleiotropy, using intercept terms to signal potential horizontal pleiotropy [14]. Additionally, we utilized the “leave-one-out” technique to exclude specific SNPs that independently influenced the MR method.

### 2.5 Multivariate analyses

Multivariate analyses were conducted using multivariable Mendelian randomization to account for potential confounders such as BMI, smoking, and alcohol use frequency. SNPs associated with these factors were included in the analysis, and causal estimates were obtained using the IVW method. Sensitivity analyses, including MR-Egger regression and the weighted median method, confirmed the robustness of the results and minimized the influence of pleiotropy.

### 2.6 Colocalization analysis

To further validate the results of MR and explore the genetic connections between asthma and AS, we conducted Bayesian colocalization (COLOC) analysis based on GWAS summary statistics of asthma and AS. COLOC analysis, a statistical method rooted in GWAS data, employs single-variant summary statistics to assess whether two independently associated genetically correlated traits share a common genetic locus. We conducted this statistical analysis using R version 4.3.3 and the “coloc” package.


**Informed consent:** Written informed consent from the participants’ legal guardians or next of kin was not required by national legislation and institutional guidelines for participation in this study.
**Ethical approval:** Ethical review and approval were not required for this study on human participants according to local legislation and institutional guidelines.

## 3 Results

### 3.1 Univariable MR estimates

**Figure 2:**
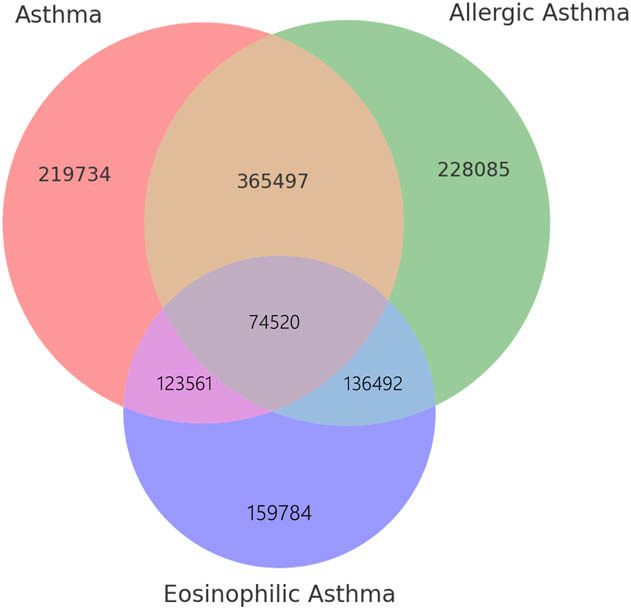
Venn diagram showing overlap between asthma-related traits. As, ankylosing spondylitis; IVW, inverse variance weighted; OR, odds ratio; Cl, confidence interval; a statistically significant (*P* < 0.05).

**Figure 3:**
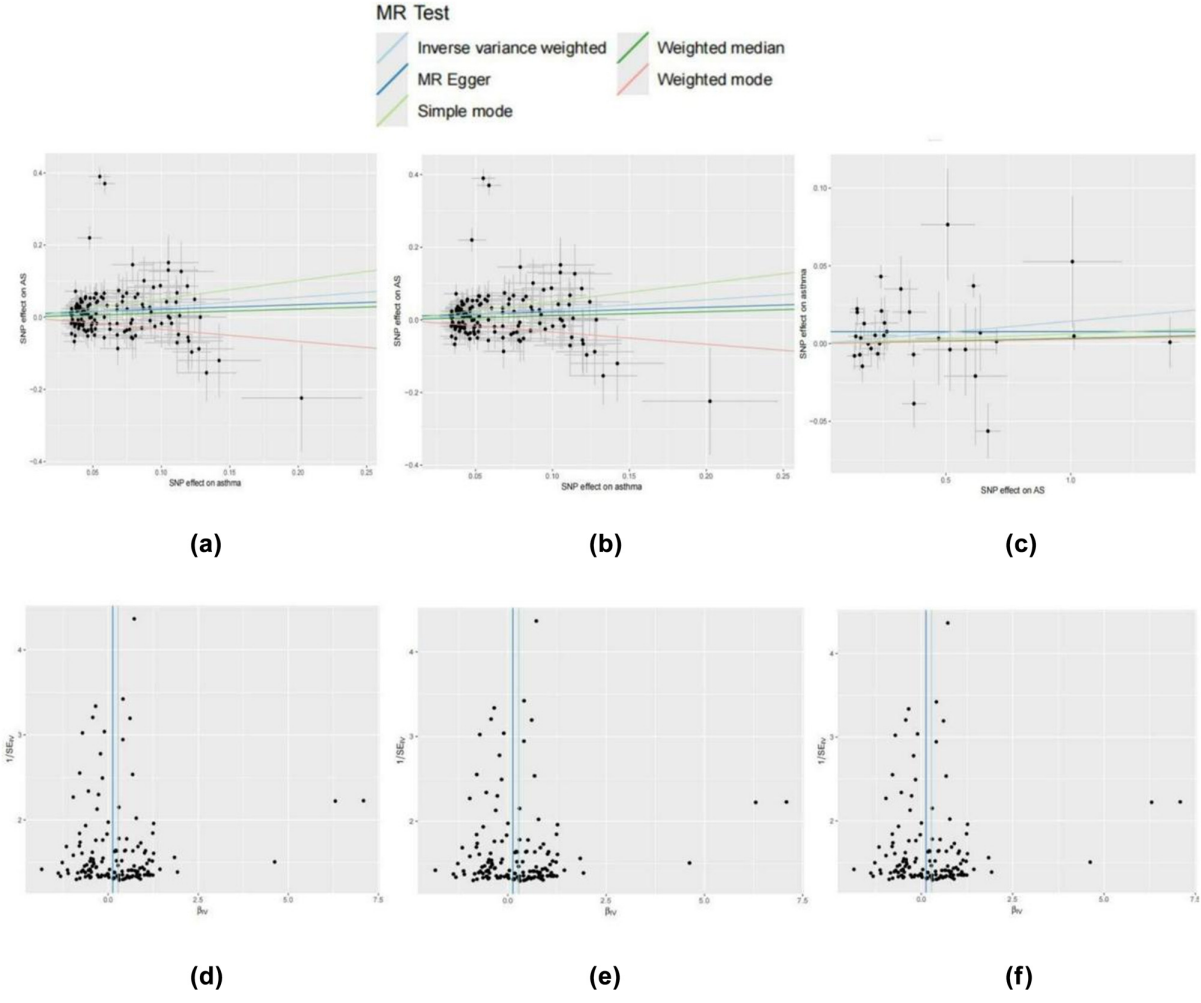
Scatter and funnel plots of the results of Mendelian randomization. (a) Scatterplot with asthma as the exposure, while As as the outcome. (b) Scatterplot with eosinophilic asthma as the exposure, while As as the outcome. (c) Scatterplot with As as the exposure, while asthma as the outcome. (d) Funnel plot asthma as the exposure, while As as the outcome. (e) Funnel plot with eosinophilic asthma as the exposure, while As as the outcome. (f) Funnel plot with As as the exposure, while asthma as the outcome.

In traditional observational studies, associations between asthma and AS have been explored through regression models, but these studies often face limitations related to confounding, reverse causality, and selection bias. A typical traditional association study might report an OR for the association between asthma and AS in the range of 1.1–1.3, suggesting a modest correlation between the two conditions. However, traditional studies often cannot rule out the possibility of reverse causality, where AS could influence the development of asthma or its symptoms. Our investigation employing the random-effects IVW model produced valuable insights. We discovered that the causal link between asthma and AS differed across the five MR techniques. Individuals with asthma and eosinophilic asthma exhibited a relatively higher risk of AS (asthma: OR 1.31, 95% CI 1.07–1.62, *P* = 0.008; eosinophilic asthma: OR 1.24, 95% CI 1.005–1.544, *P* = 0.044). For allergic asthma, childhood-onset asthma, and obesity-related asthma, all five MR methods showed no causal relationship with AS (Figure 2). The scatterplot shows that each point represents an IV, with the lines on each point indicating the 95% confidence interval (CI) (Figure 3a–c). The colored lines represent the results of the MR fitting. Patients with asthma and eosinophilic asthma have a relatively higher risk of developing AS. The funnel plot results are consistent with these findings, aligning with the results mentioned above (Figure 3d–f).

**Figure 4:**
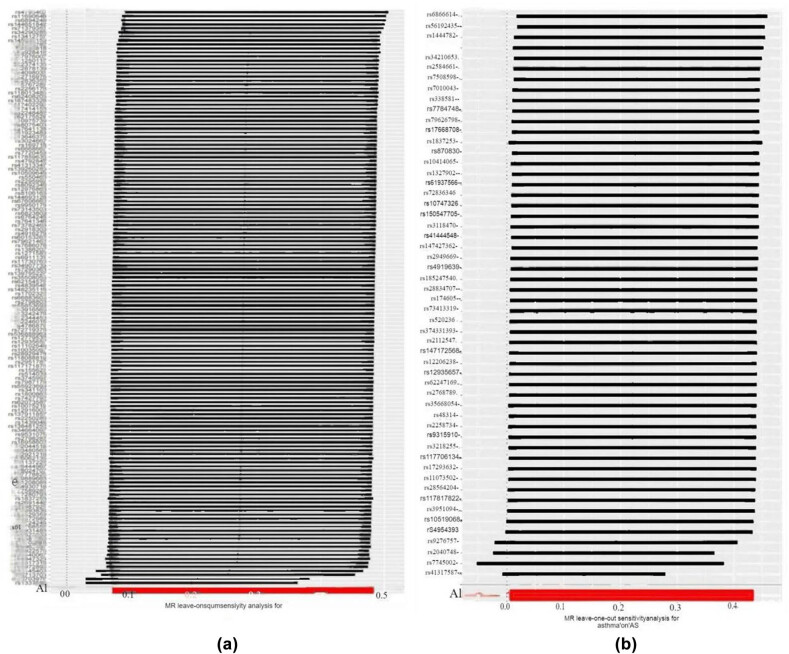
The results of leave-one-out analyses: (a) Asthma on AS and (b) eosinophilic asthma on AS.

For sensitivity analysis, heterogeneity was detected in the MR analysis among asthma, eosinophilic asthma, and AS (asthma *P* = 1.03 × 10^−67^; eosinophilic asthma *P* = 2.59 × 10^−234^) (Table 2). The MR-Egger analysis indicated no significant horizontal pleiotropy (*P* = 0.57). Although we observed some heterogeneity in our analysis, this heterogeneity had less impact on our results because we used a random effects model. Additionally, the leave-one-out analysis, where each SNP is sequentially removed, further demonstrated a robust causal relationship, as no significant difference in effect estimates was observed before and after removal (Figure 4a and b).

**Table 2:** Heterogeneity and pleiotropy analysis between asthma and ankylosing spondylitis

Exposure	Outcome	Heterogeneity		Pleiotropy			
		*Q*	*Q* df	*P*-value	Egger intercept	SE	*P*-value
Asthma	AS	659	142	1.03 × 10^−67^	0.009	0.015	0.57
Eosinophilic asthma	AS	1,268	53	2.59 × 10^−234^	0.02	0.06	0.7
Obesity-related asthma	AS	132	47	4.60 × 10^−10^	0.015	0.02	0.5
Allergy-related asthma	AS	365	66	4.02 × 10^−43^	0.03	0.02	0.2
Childhood-onset asthma	AS	1,173	54	4.37 × 10^−210^	0.02	0.05	0.67
AS	Asthma	104	33	2.07 × 10^−09^	0.007	0.005	0.15

### 3.2 Reverse-direction MR analysis

**Figure 5:**
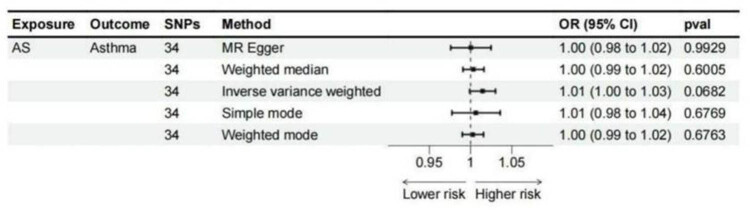
Reverse-direction MR analysis. Each point represents an individual genetic variant, with the *Y*-axis indicating the effect estimate (odds ratio, OR) and the *X*-axis the SEs. The colored lines show the 95% CI.

To explore the relationship between AS and asthma along with its subtypes, we carried out a reverse MR analysis. All five MR technique indicated no significant link between AS and asthma (Figure 5). Although we observed some heterogeneity in our analysis, random effects models eliminate heterogeneity risk. Sensitivity analyses indicated no potential horizontal pleiotropy (Table 2). One potential source of heterogeneity could be differences in genetic instruments used for different subtypes of asthma. Since the genetic instruments for asthma subtypes may vary in their strength or relevance to the specific asthma phenotype, it is possible that some instruments may not adequately capture the true relationship between asthma and AS. Furthermore, heterogeneity could stem from the underlying biological differences between asthma subtypes. For instance, eosinophilic asthma, allergic asthma, and childhood-onset asthma may involve distinct immunological mechanisms that could differentially influence the relationship with AS.

### 3.3 Multivariable MR estimates

**Figure 6:**
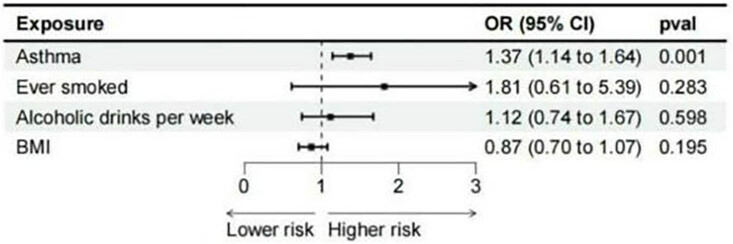
Multivariable MR estimates.

In multivariate MR analyses, we individually adjusted for cigarette smoking, alcohol consumption frequency, and BMI. The purpose of these adjustments was to increase statistical power by accounting for potential confounding factors. Multivariate MR analyses did not change the effect size substantially but provided more precise estimates (IVW OR = 1.37, *P* = 0.001) (Figure 6).

### 3.4 Colocalization between AS and OA

**Figure 7:**
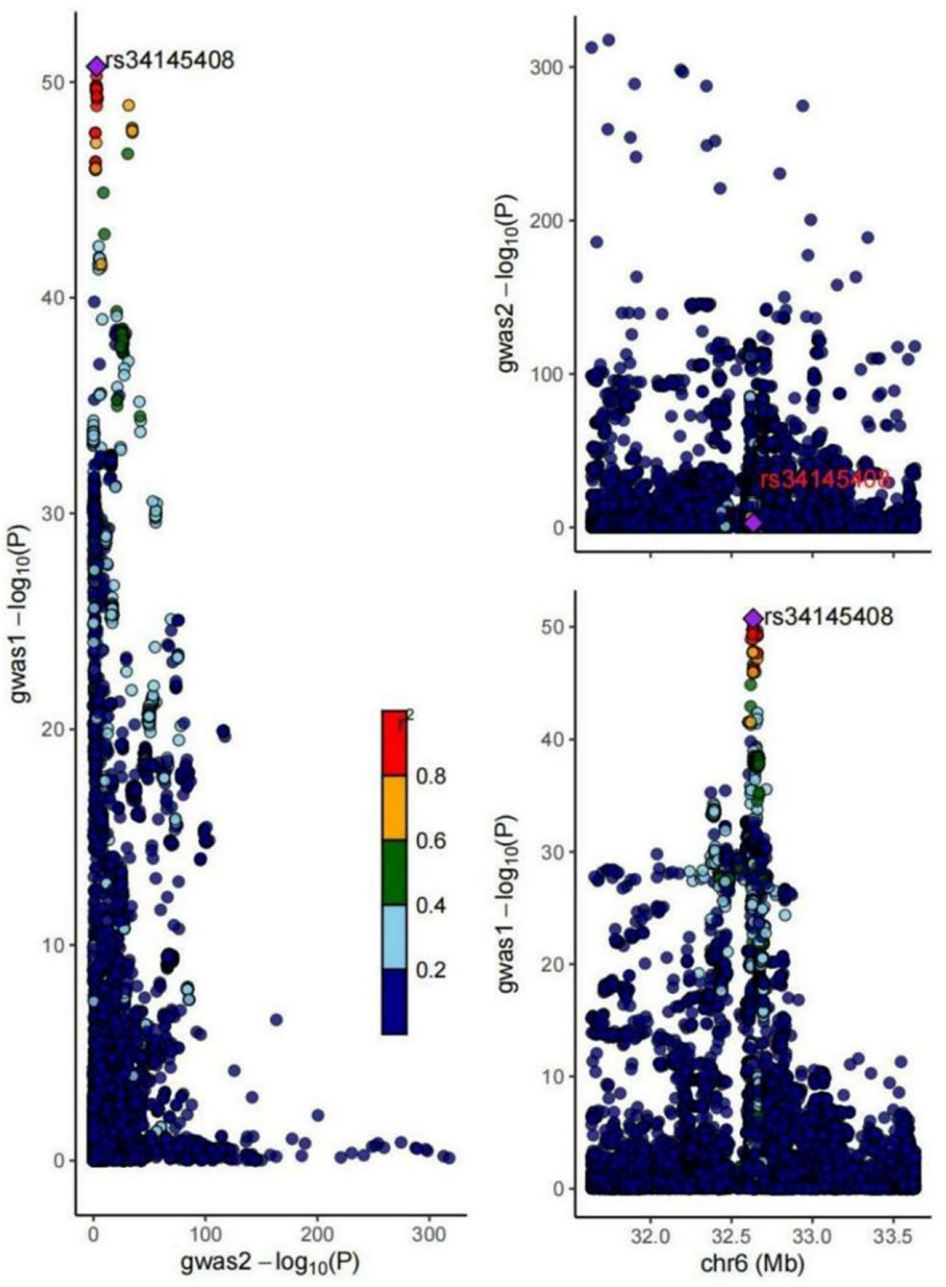
Result of colocalization between asthma and As. Each point represents a single variant, with color intensity reflecting the strength of the association between the variant and both conditions. *P*-values are indicated as follows: Red for highly significant associations (*P* < 0.01) and blue for non-significant associations (*P* ≥ 0.05).

The colocalization analysis provided minimal evidence that the two traits, which showed a causal link in the MR analysis, were colocalized (pp4 = 2.4 × 10^−46^, pp3 = 1). This implies that the SNPs in the gene locus could be in LD with nearby variants, making them unsuitable for drug target perturbation, or that multiple causal variants might be present in the region, rendering colocalization analysis inappropriate. At the same time, the PP.H3 value was notably elevated, suggesting that variants in this area were strongly linked to asthma and AS, though influenced by distinct factors (Figure 7).

## 4 Discussion

This research evaluated the two-way causal link between asthma, its various forms, and AS. Our findings, derived from a TSMR method and combined GWAS data, revealed a positive causal link between asthma and AS. The underlying mechanisms of this association remain unclear, but existing studies suggest a potential link between the two conditions. The pathogenesis of AS is not fully understood, though it is believed to involve both autoinflammatory and autoimmune mechanisms. Research involving genetics and experimental frameworks indicates that the IL-23-IL-17 pathway contributes to the development of spondyloarthritis (SpA), and IL-17 inhibitors are key components of modern therapies [15]. Asthma, marked by inflammation driven by type 2 helper T (Th2) cells and cytokines like IL-4, IL-5, and IL-13, also involves Th17 cells and IL-17 in more severe instances [16]. Asthma and AS may share inflammatory mediators and pathways, such as TNF-α and IL-17. Additionally, autoimmunity is considered a key pathological mechanism in intrinsic asthma, with the co-occurrence of allergy and autoimmunity, as well as the presence of autoantibodies, supporting the hypothesis of asthma autoimmunity [17–19]. This suggests that asthma may increase the risk of AS through abnormal immune system regulation. We also identified a significant causal link between eosinophilic asthma and AS, which has been further supported by another research team uncovering potential biological mechanisms. One study highlighted a case series of five patients with severe asthma who were simultaneously diagnosed with rare autoinflammatory conditions, including AS [20]. While eosinophilic asthma primarily targets the airways and AS affects the joints, both conditions may share a common underlying issue – immune system dysfunction, involving a breakdown in immune tolerance and excessive activation of immune pathways, such as inflammasome activation. This shared immune dysregulation could explain the stronger association between eosinophilic asthma and AS compared to other asthma types. Further research is needed to better understand the genetic and immune mechanisms linking these diseases.

Additionally, research has shown that asthma and AS might share common genetic factors. HLA-B27 is a recognized risk gene for AS, with genetic analyses revealing that this gene within the major histocompatibility complex region contributes to about 20.1% of AS heritability, with 4.3% linked to other loci beyond HLA-B [21]. Interestingly, certain HLA gene variants such as HLA-DQA1, HLA-DQB1, and HLA-DRB9 are also associated with asthma [22,23]. This gene sharing may explain the causality observed in MR analysis. However, no loci common to both diseases have been identified yet. This result might indicate complex genetic architecture, where distinct genetic mechanisms are simultaneously influencing the two traits, or it could reflect methodological limitations of the colocalization approach when applied to polygenic traits. Future studies using higher-resolution data or alternative approaches may help clarify these findings. Additional research is required to investigate this possible explanation. Additionally, several environmental factors could explain the association between asthma and AS. For instance, smoking can elevate inflammation in the lower airways through multiple processes and serves as a risk factor for asthma [24]. Rom et al. found that smokers exhibited higher levels of acute-phase C-reactive protein, white blood cell counts, and pro-inflammatory cytokines such as TNF-α and IL-6 compared to non-smokers [25]. Smoking has also been shown to activate inflammatory cells through the NF-κB pathway, promoting the differentiation and activation of Th17 cells, which are closely associated with AS development [26,27]. Thus, smoking is recognized as a shared risk factor. Additionally, our findings align with these hypotheses. However, reverse MR analysis revealed no causal link between AS and asthma, contrasting with a retrospective cohort study on the Taiwanese population conducted by Shen et al. This discrepancy may be related to several factors, including differences in study populations, such as genetic and environmental factors that may vary between the Taiwanese and European populations. Additionally, methodological variations, such as differences in study design, data collection, or statistical approaches, could also contribute to the contrasting findings. Further verification with larger sample sizes and more comprehensive inclusion of diverse ethnic groups is needed to address these potential confounders and strengthen the robustness of the conclusions. A key advantage of this research lies in employing MR analysis, which utilizes genetic variation as an IV to deduce causation, thereby effectively mitigating biases from reverse causality and confounding variables. However, there are limitations to MR analysis that must be acknowledged. First, our MR analyses focused on European populations to minimize racial confounding. Therefore, there is an urgent need for dependable data on non-European or diverse populations to explore connections with particular genes influenced by the local environment. Second, despite the MR design and the exclusion of known confounders, unaccounted potential confounders may still affect the results. Additionally, susceptibility to autoimmune diseases is generally higher in women than in men. However, we were unable to stratify the study by sex due to limitations in the available data from the original GWAS. Additionally, this research explored the connection between various forms of asthma and AS. However, due to the quality of the GWAS samples, the analysis was limited to eosinophilic asthma, allergic asthma, childhood-onset asthma, and obesity-related asthma. While the outcomes provide a basis for further research, they are not comprehensive enough.

## 5 Conclusion

Overall, this study found a significant causal relationship between asthma, particularly eosinophilic asthma, and AS, while no substantial causal link was identified between allergic asthma, childhood-onset asthma, or obesity-associated asthma and AS. The results were derived from MR analyses, which provided strong evidence for the association between asthma subtypes and an increased risk of AS. Future research should focus on addressing the gaps in the causal chain between asthma and AS, which could include in-depth molecular biology studies and animal model experiments. Furthermore, extensive, prolonged cohort studies are essential to confirm MR analysis findings and investigate other possible shared risk factors.
